# Long-term trends of pediatric type 1 diabetes incidence in Japan before and after the COVID-19 pandemic

**DOI:** 10.1038/s41598-023-33037-x

**Published:** 2023-04-10

**Authors:** Fumika Matsuda, Tomoyo Itonaga, Miwako Maeda, Kenji Ihara

**Affiliations:** Department of Pediatrics, Oita University Faculty of Medicine, 1-1 Idaigaoka, Hasama, Yufu, Oita 879-5593 Japan

**Keywords:** Type 1 diabetes, Epidemiology, Paediatric research

## Abstract

Type 1 diabetes incidence has increased worldwide, although the long-term trends on pediatric type 1 diabetes in Japan remain elusive. To investigate the incidence and secular trend of pediatric type 1 diabetes from 1999 to 2021, including the coronavirus disease 2019 (COVID-19) pandemic years, in Oita Prefecture, Japan. We investigated the number of newly diagnosed patients with type 1 diabetes aged < 15 years from 1999 to 2021. We surveyed hospital databases in Oita Prefecture in Japan. The type 1 diabetes incidence in children aged < 15 years increased annually by 5.3% among all children, especially in boys aged 10–14 years by 8.1%, over the past 23 years. The average incidence rate of 3.9/100,000/year was nearly consistent with the previous reports on Asian countries. No significant change was found in the increasing incidence trend of type 1 diabetes before and during the COVID-19 pandemic. The incidence of pediatric type 1 diabetes has significantly increased over the past 23 years in Oita Prefecture, Japan, which is consistent with the worldwide trend.

## Introduction

Type 1 diabetes (T1D) is an autoimmune disease characterized by insulin deficiency and subsequent hyperglycemia^1^. T1D is classified as a polygenic disease with identical twin concordance of 30–70%^2^, sibling risk of 6–7%, and a 1–9% risk for children who have a parent with T1D^3^. Ethnicity deeply contributes to the incidence of T1D. The surveillance in the United States (US) demonstrated that Caucasians are more susceptible to T1D than African- and Hispanic-Americans, whereas Asians, including Chinese, Korean, or Japanese, and individuals from South America were the least susceptible^4^. According to data from the International Diabetes Federation Atlas 2022 Reports, 8.75 million individuals had T1D worldwide and 530,000 patients were newly diagnosed among all age groups in 2022, 201,000 of whom were < 20 years of age^5^. The gradual increase in the incidence of T1D in children < 15 years old is reported as a worldwide trend^6^. In the US, children and adolescents aged < 20 years showed annual increase at 1.8% from 2002 to 2012^7^, with a similar increase of 1.3% reported for the Canadian province of British Columbia for the period of 2002–2013^8^. In Australia, a significant increase of 1.2% (95% confidence interval [CI] 0.4%, 2.1%) was observed in the 10- to 14-year-old age group during the 2000–2011 period^9^. Among European countries, no increase was found in Sweden during the period from 2005 to 2007^10^, and similarly flattening incidence rates were subsequently reported in two other high-incidence Scandinavian countries (Finland^11^ and Norway^12^). Asian countries with extremely low incidences of T1D (0.4–1.1 cases/100,000 individuals/year), such as Uzbekistan, Korea or China, also have increasing trends of T1D (3–12% increase)^13–15^. However, no remarkable increase has been reported in Japan. For example, Kawasaki et al. reported T1D incidence in patients aged 0–14 years averaging 2.37 cases/100,000 individuals/year from 1993 to 2001^16^. Onda et al. also reported a minimal increase in the T1D incidence in Japan from 2.24 (1998–2001) to 2.27 (2005–2010) cases per 100,000 individuals/year^17^. However, the results seemed elusive, partly because some of the newly diagnosed patients might have been omitted in the registration system in Japan, called Medical Aid Program for Chronic Pediatric Diseases of Specified Categories (MAPChD)^18^. Since a nationwide all-encompassing registration system for children with T1D has not been established in Japan, the MAPChD data would be insufficient for a national epidemiological study. Alternatively, the local registry systems in each prefecture or small-scale adjacent areas would be better for extracting the epidemiological data about patients with T1D. For instance, a recent study in Yamanashi Prefecture in Japan reported that a significant increase was not observed from 1986 to 2018 with an annual rate of increase at 1.16%; among them, the subpopulation aged 5–9 years had a significant annual rate of increase at 5.38% (CI 2.34–8.35%)^19^.

The large difference observed in the incidence rates of T1D among ethnic groups is primarily derived from the genetic characteristics in susceptible or resistant haplotypes of the human leukocyte antigens (HLA) gene^6^. Additional genetic factors also influence susceptibility to viral infections or following provocation of autoimmunity against pancreatic beta cells^20–22^. Therefore, the recent changes in the prevalence of T1D among Japanese children may differ from those in other countries depending on epidemic infectious diseases, including the SARS-CoV-2.

The present study aimed to explore the incidence, prevalence, and annual trend of childhood-onset T1D in Oita Prefecture, which is a representative rural area of Japan. We precisely investigated the trends over time in the incidence of T1D in children < 15 years. To avoid missing any cases, we employed three different strategies of data collection. First, the clinical information of newly diagnosed patients with T1D was extracted from the medical records of three core hospitals in Oita Prefecture. Second, we directly inquired with pediatricians at all domestic hospitals for children in Oita Prefecture. Third, the MAPChD data were carefully applied to confirm the accuracy of the epidemiological data. Finally, based on the annual trend of childhood-onset T1D, we focused on the possible change in the incidence of pediatric T1D during the COVID-19 pandemic years in Japan.

## Results

**Figure 1:**
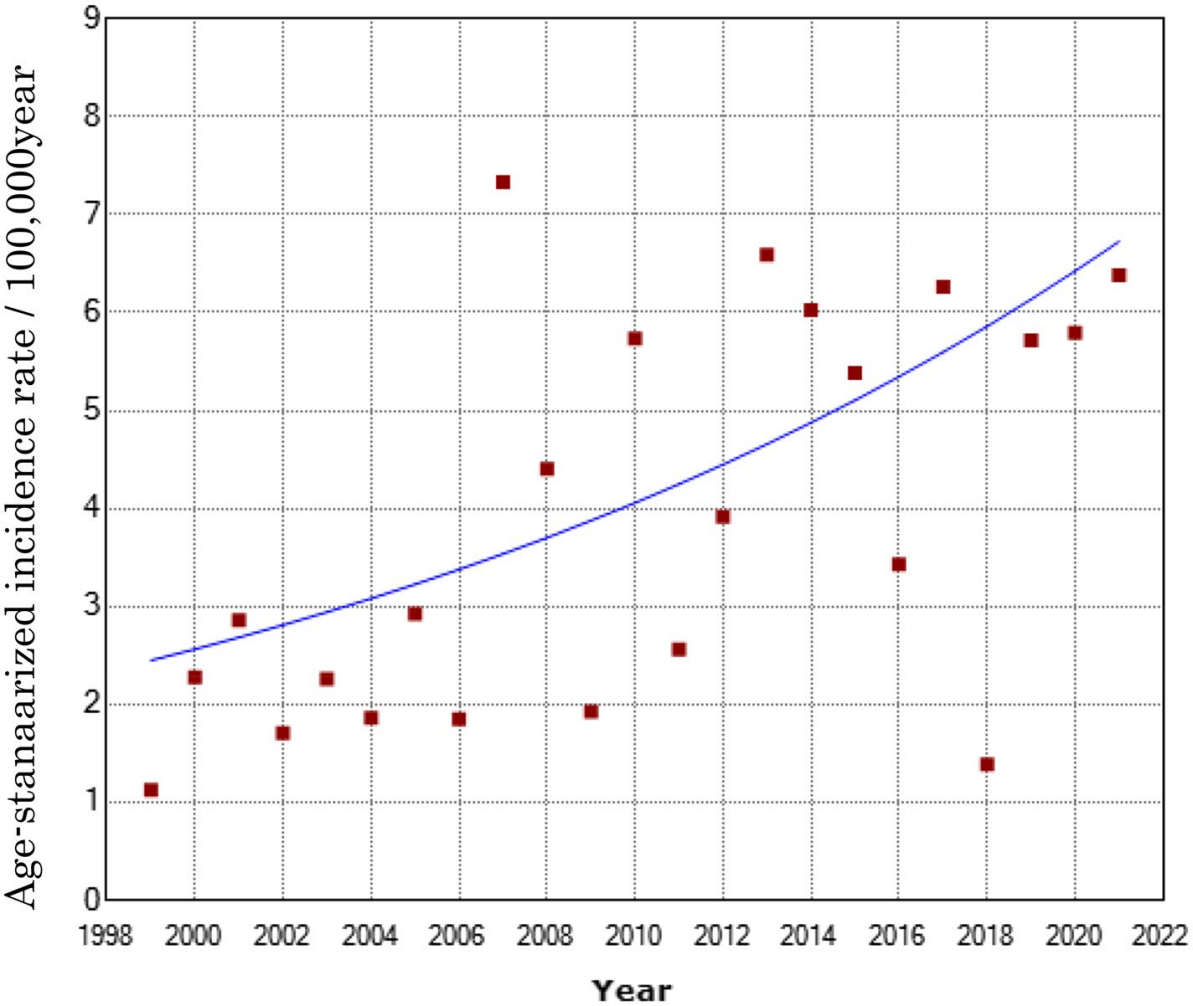
Annual trend of the age-standardized incidence rate of Type 1 Diabetes (T1D) aged 0-14 years during 1999–2021. The incidence of type 1 diabetes in those aged ≤ 14 years increased annually by 4.7% (P = 0.004; 95% CI [1.7, 7.8]).

From January 1999 to December 2021, 137 children and adolescents were newly diagnosed with T1D in Oita Prefecture, Japan (Table 1) including 67 girls and 70 boys. The overall incidence rate in the study period was 3.9/100,000 individuals/year. For the total cohort (aged ≤ 14 years), a significant constant increase was observed (annual percent change [APC], 4.7; 95% CI 1.7, 7.8) (Fig. 1 and Supplementary Table). When incidences were compared between boys and girls, the age-standardized incidence rate of T1D in boys statistically increased during the 20-year study period (APC, 6.2; 95% CI 1.3, 11.4) (Table 2). When incidences were stratified by sex and age groups (0–4, 5–9, and 10–14 years), the age-standardized incidence rate of T1D in boys (10–14 years old) statistically increased during the 20 years (APC 8.1, 95% CI 3.2, 13.2) (Table 2). A sharp increase was observed in 2007, 2013, and 2017 (7.44, 6.60, and 6.60/100,000 individuals/year, respectively), although no cyclic occurrence by the stratification of 4-year intervals (1999–2002, 2003–2006, 2007–2010, 2011–2014, and 2014–2018) (Supplementary Table).

**Table 1:** Numbers of type 1 diabetes cases and incidences (/1000,000 year) among children of each age group during 1999–2021.

	Year	1999	2000	2001	2002	2003	2004	2005	2006	2007	2008	2009	2010	2011	2012	2013	2014	2015	2016	2017	2018	2019	2020	2021
0–4 years	Cases	0	3	1	1	0	3	0	1	2	2	1	4	2	2	2	2	3	1	2	0	1	2	5
	Incidence rate	0	5.40	1.83	1.85	0	5.69	0	1.99	4.00	3.98	1.98	7.89	4.03	4.04	4.08	4.11	6.26	2.21	4.50	0	2.35	4.80	12.97
5–9 years	Cases	2	0	3	0	0	0	1	1	4	4	2	2	0	2	4	5	1	1	4	1	5	2	2
	Incidence rate	3.37	0	5.18	0	0	0	1.82	1.83	7.38	7.53	3.83	3.92	0	4.01	8.06	10.13	2.02	2.03	8.20	2.09	10.7	4.40	4.35
10–14 years	Cases	0	1	1	2	4	0	4	1	6	1	0	3	2	2	4	2	4	3	3	1	2	4	1
	Incidence rate	0	1.51	1.57	3.23	6.66	0	6.83	1.73	10.49	1.76	0	5.45	3.67	3.70	7.56	3.84	7.83	5.98	6.05	2.03	4.07	8.12	2.03

**Table 2:** Annual increases in incidence rate for 20-year period (1999–2018) as estimated by mixed effects Poisson regression in subgroups defined by age group and sex.

Subgroup		Annual increase (%)	(95% CI)	P-value	Combination
Boys	0–4 years old	− 1.4	(− 10.6, 8.9)	0.688	Every 4 years
	5–9 years old	8.0	(− 1.2, 18.1)	0.071	Every 4 years
	10–14 years old	8.1	(3.2, 13.2)	0.013*	Every 4 years
	0–14 years old	6.2	(1.3, 11.4)	0.018*	Every 2 years
Girls	0–4 years old	7.6	(− 12.2, 31.9)	0.262	Every 5 years
	5–9 years old	4.2	(− 18.0, 32.3)	0.625	Every 4 years
	10–14 years old	2.7	(− 6.8, 13.1)	0.448	Every 4 years
	0–14 years old	4.8	(− 2.7, 12.9)	0.181	Every 2 years
Total	0–4 years old	3.1	(− 1.9, 8.2)	0.198	Every 2 years
	5–9 years old	4.9	(− 10.7, 23.4)	0.412	Every 4 years
	10–14 years old	5.0	(− 0.7, 10.9)	0.079	Every 2 years
	0–14 years old	5.3	(1.1, 9.8)	0.016*	Every 1 years

The cohort is not processed if some of the records for a cohort have zero counts. We aggregated the data by combining some of the years in order to eliminate the zero values according to the software operation manual. We combined the data in the smallest units.

*P < 0.05.

We explored whether the COVID-19 pandemic altered the occurrence of new-onset T1D. We compared the annual increase in the incidence of T1D during 20 years prior to the COVID-19 pandemic (1999–2018) and that during 23 years including the pandemic period (1999–2021). As the result, the annual increases of these periods were almost the same (5.3 and 4.7, respectively). We concluded that no significant increasing was observed in the T1D prevalence during the COVID-19 outbreak in Oita Prefecture, Japan.

## Discussion

Our study demonstrated that the incidence of T1D in children < 15 years in Oita Prefecture, a rural area in Japan, has been significantly increasing every year by 4.7% during the past 23 years (1999–2021). The increasing trend of T1D incidence in Oita Prefecture was almost consistent with the previous report from Asian countries^14^. The average incidence rate of 4.7/100,000 individuals/year in Oita Prefecture was higher than that of a previous report conducted in Yamanashi Prefecture (2.0/100,000 individuals/year; 1988–2016)^19^ located in the middle of Honshu Island and surrounded by mountains with a population of approximately 800,000 residents. The Japanese population is classified as a nearly single race country with more than 95% of the Yayoi people. T1D is recognized as a multifactorial disease and therefore local and specific environmental factors, including viral outbreaks, play important roles in the development of T1D. Our data from Oita Prefecture was slightly different from those of Yamanashi. A difference in environmental factors may exist between Yamanashi and Oita Prefectures; however, the cause remains unknown. In an Asian population, a report from Zhejiang province in the low-incidence region of China described a rapid annual incidence increase rate of 12.0% among those aged < 20 years from 2007 to 2013^15^. The incidence and prevalence of childhood-onset T1D in Korea from a nation-based registry demonstrated an increase of 3–4% every year from 2007 to 2017. In Korea, the overall incidence of childhood-onset T1D increased from 3.70% in 2008 to 4.77% in 2016, according to the Health Insurance Review and Assessment Service^14^. The causes for this ethnicity-independent T1D increase also remain unelucidated. The increase in T1D incidence rates in Korea are considerably higher than those in other countries. Changing of the populations’ genetic pool was unlikely to affect the increasing trend. However, local or regional environmental factors might have impacted the increasing trend of pediatric T1D. A further nationwide study is needed to evaluate the association between the occurrence of T1D and types of viral infection.

Many groups have reported the T1D incidence in children during the COVID-19 pandemic. A meta-analysis estimated the global risk of new onset pediatric patients with T1D before and after the COVID-19 pandemic, and reported the incidence rates of 19.73 and 32.39 per 100,000 children in 2019 and 2020, respectively^23^. Compared with the incidence before COVID-19 pandemic, new-onset pediatric T1D during the first year of the COVID-19 pandemic increased by 9.5% worldwide^23^. In Europe, the remarkable increase in T1D incidence observed in the pediatric populations of Calabria (southern Italy) or Piedmont (northwest Italy) might be related to the global impact of the COVID-19 pandemic from 2019 to 2021^24^. Similar increasing trends were observed in Romanian and Spanish children in 2020^25,26^. In contrast, an increase in T1D incidence among people aged ≤ 20 years was not found during the COVID-19 pandemic (3/2020–12/2021) in Germany^27^. Many other studies have attempted to analyze whether the COVID-19 pandemic affected the incidence of T1D, nevertheless conflicting results have been shown from various reasons. For example, symptoms can vary with COVID-19, and children are often asymptomatic or mildly affected compared with adults. The antigen test or even the polymerase chain reaction method for SARS-CoV-2 detection is imperfect, therefore a negative test result does not exclude a previous mild or asymptomatic infection. In addition, substantial biases of genetic susceptibility or heterogeneity in the latency from the time of the infection to diabetes onset might have existed in the studied populations. Moreover, awareness of diseases might be inconsistent caused by lifestyle changes during and after lockdowns. The COVID-19 pandemic drastically changed hygiene practices and social distancing, which reduced the incidence of other viral infections, especially in children. In addition, SARS-CoV-2 strains continue to mutate over time, resulting in genetic variations among the circulating viral strains, which reduce symptom severity although greatly increase the infection rates in children. Therefore, these environmental changes on the host side and pathogenic alterations on the viral side could have affected the T1D incidence in children. Therefore, long-term studies should be conducted to investigate the direct and indirect effects of COVID-19 infections on the development of T1D, and would be helpful to understand the pathogenesis and make prevention of T1D.

No significant change in the T1D occurrence before and during the COVID-19 pandemic was observed in our data; however, this study had some limitations. The sample size was relatively small compared with those in previous studies in other countries. The two years of survey during the COVID-19 pandemic might be too short to conclude the results. In particular, COVID-19 rates in children have been drastically increasing since the beginning of 2022 in Japan; therefore, further study after 2022 will be essential to clarify the direct impact of COVID-19 on the development of T1D. In addition, people avoided visiting doctors' offices or hospitals in afraid of COVID-19 infection during the epidemic period and it might have influenced the results. Despite these limitations, our preliminary study provides basic information on the incidence and prevalence of pediatric T1D during the COVID-19 pandemic in Japan.

In conclusion, the incidence of pediatric T1D in Oita Prefecture, a rural area of Japan, has significantly increased over the past 23 years, consistent with the worldwide trend. No significant increasing trend was observed during the past 2 years during the COVID-19 pandemic.

## Methods

### Geographic features of Oita Prefecture

Oita Prefecture is located on the northeast side of the coastal area of Kyushu Island. Oita Prefecture has a population of 1,124,983 (October 1, 2020) and a geographic area of 6340 km^2^. Oita faces the seaside and is surrounded by mountains. It consists of 18 municipalities, including Oita City. Oita City, the capital of Oita Prefecture, is located in the east center of Oita Prefecture of the coastal area, with a population of approximately 470,000 in 2020, accounting for 40% of the prefecture's population. The population of Oita Prefecture had been steadily decreasing year by year. Children aged < 15 years and older individuals aged > 65 years account for 12.1% and 33.3% of the population, respectively. The corresponding numbers of the national average in Japan are 11.9% and 28.6%, respectively. Therefore, Oita Prefecture mimics a roughly a 1/100 scaled-down version of Japanese society in the rural area.

### Study population

Patients newly diagnosed with T1D who were < 15 years of age and living in Oita Prefecture were enrolled in this study from January 1999 to December 2021.

Three strategies of data collection methods were prepared for this study. First, clinical information of both inpatients and outpatients with newly diagnosed T1D was extracted from the medical records in three core hospitals: Oita University Hospital, Oita Prefectural Hospital, and National Hospital Organization Nishi-Beppu National Hospital. After the approval of this clinical study in the enrolled hospitals, we directly asked the pediatricians in the domestic hospitals by phone or e-mail about the patient's medical information including gender, date of birth, age at onset, and the objective evidence for the T1D diagnosis such as the presence of GAD/IA2 antibodies or with or without ketosis, or types of insulin treatment during the acute phase. The information was provided in a deeply sensitive manner, such as by not providing their names, address information, or patient ID numbers. Second, the pediatricians in all domestic hospitals were directly asked questions regarding facilities for children in Oita Prefecture as follows: Nakatsu Municipal Hospital, Kunisaki Municipal Hospital, Bungo-Ono Municipal Hospital, Saiseikai Hita Hospital, and Tsurumi Hospital. Third, the patients with confirmed T1D registered in MAPChD database were included. The MAPChD records in Oita Prefecture are separately stored under the management of two government offices: the Oita City Government for Oita City citizens and the Oita Prefecture for citizens of all cities and towns, except for Oita City. Since the Oita City data before 1999 and those of Oita Prefecture before 2009 were not stored, we used the Oita City data from 2000 to 2021 and those of Oita Prefecture from 2010 to 2021. Hence, from 2000 to 2009, the MAPChD data only from Oita City was used for the validation of the information about T1D patients. As for the T1D patients of the Oita Prefecture outside Oita City during this period, we carefully collected data on newly onset T1D patients in the hospitals, since they were continuously managed in these hospitals. Therefore, there would be little, if any, unregistered T1D patients during this period, whereas the data from three independent sources facilitated to find newly onset T1D patients as entirely as possible.

The population statistics in Oita Prefecture were referred from the database of vital statistics in Oita Prefecture (https://www.pref.oita.jp/site/toukei/index-cpe.html).

### Statistical analysis

Incidence rates were calculated by dividing the numbers of registered children by annual population estimates in Oita Prefecture, Japan. Estimates of rate increases were obtained using a mixed effects Poisson regression model with age and sex as fixed effects. Time trends of age-standardized rates and APC and P-values were estimated by Joinpoint analysis (Joinpoint Regression Program, Version 4.9.; Statistical Research and Applications Branch, National Cancer Institute, Bethesda, MD, US). The estimated annual rate of change in the prevalence was calculated using ln(y) = xb drawn by taking the natural logarithm. To calculate rates, the denominator values (i.e., number of boys and girls aged < 15 years) were obtained from the Japanese Model Population. Subgroup analyses were performed according to sex and age group in patients with T1D. When the patients were stratified by age at diagnosis, 40 patients were aged ≤ 4 years, 46 were 5–9 years, and 51 were 10–14 years. The 95% CI were also calculated for proportions. All other calculations were performed using R version 3.5.2 (R Foundation for Statistical Computing, Vienna, Austria [https://www.R-project.org/]). Rates are given per 100,000 person/year. The significance level was set at 5% for two-sided tests.

The cohort was not processed if some of their records had zero counts. We aggregated the data by combining the years to eliminate the zero values according to the software operation manual. We combined the data in the smallest units. In the as-group analysis by age and sex, data binding was required up to every 5 years. Therefore, data for 23 years was obtained, although the analysis by age and sex was for the first 20 years.

### Ethics declarations

This study was conducted in accordance with the tenets of the Declaration of Helsinki and all relevant guidelines and regulations and was approved by the ethics committee of Oita University Hospital, Oita, Japan (No. 2118). The study information was disclosed on the website (https://www.med.oita-u.ac.jp/hospital/kenkyu-rinri/index.html), and informed consent was obtained using an opt-out method. Patients who elected to opt out were excluded from this study.

## Supplementary Information

Supplementary Information.

## Data Availability

The datasets used and analysed during the current study available from the corresponding author on reasonable request.
