# Supplementary material for: Long-term trends of pediatric type 1 diabetes incidence in Japan before and after the COVID-19 pandemic
Source: Sci Rep. 2023 Apr 10;13:5803. doi: 10.1038/s41598-023-33037-x (PMC10085994; doi:10.1038/s41598-023-33037-x)
Supplement: Supplementary file 1 — Supplementary Information. [file 41598_2023_33037_MOESM1_ESM.pptx]

## Slide 1
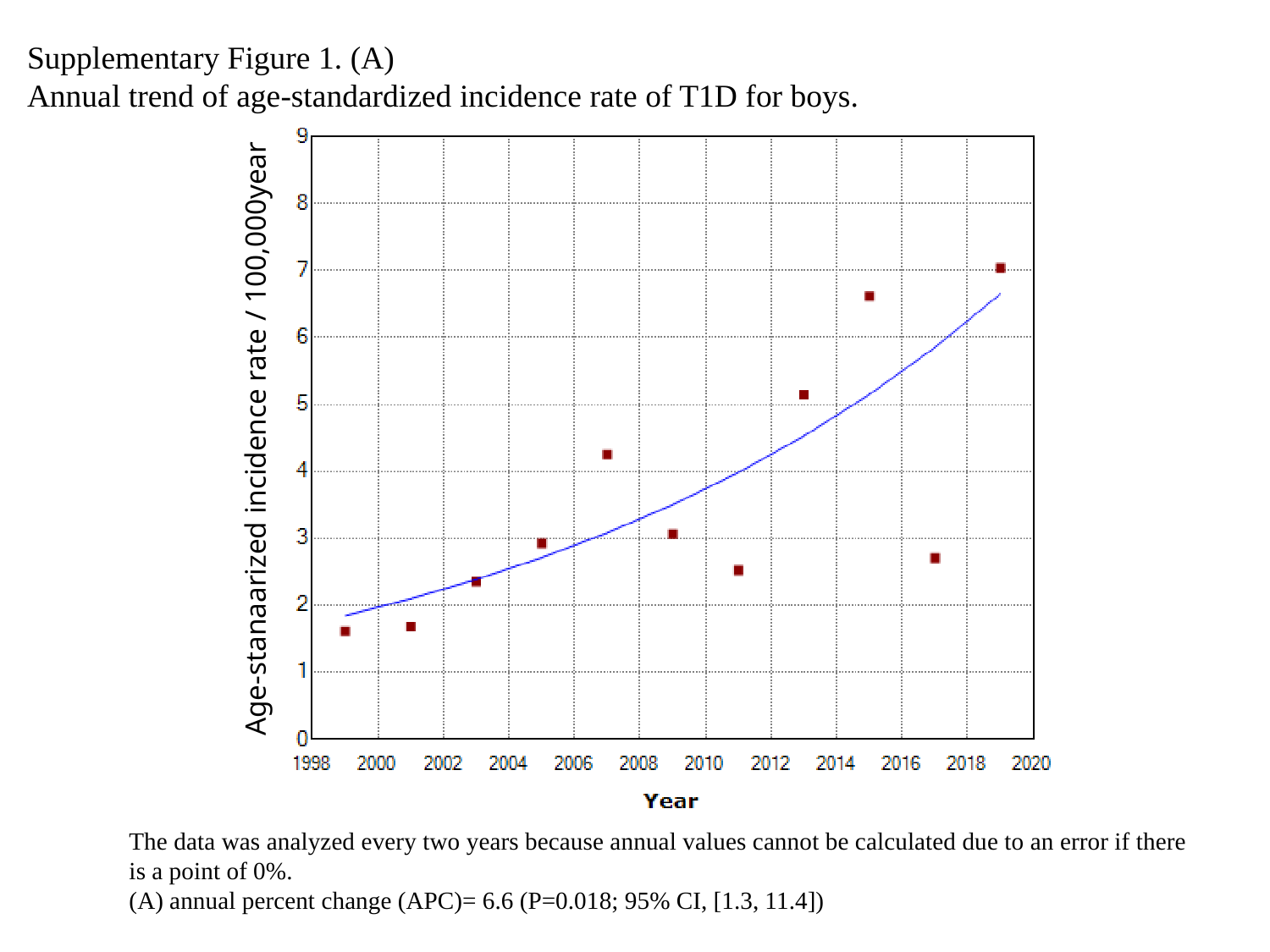

Supplementary Figure 1. (A)
Annual trend of age-standardized incidence rate of T1D for boys.
Age-stanaarized incidence rate / 100,000year
The data was analyzed every two years because annual values cannot be calculated due to an error if there is a point of 0%.
(A) annual percent change (APC)= 6.6 (P=0.018; 95% CI, [1.3, 11.4])

## Slide 2
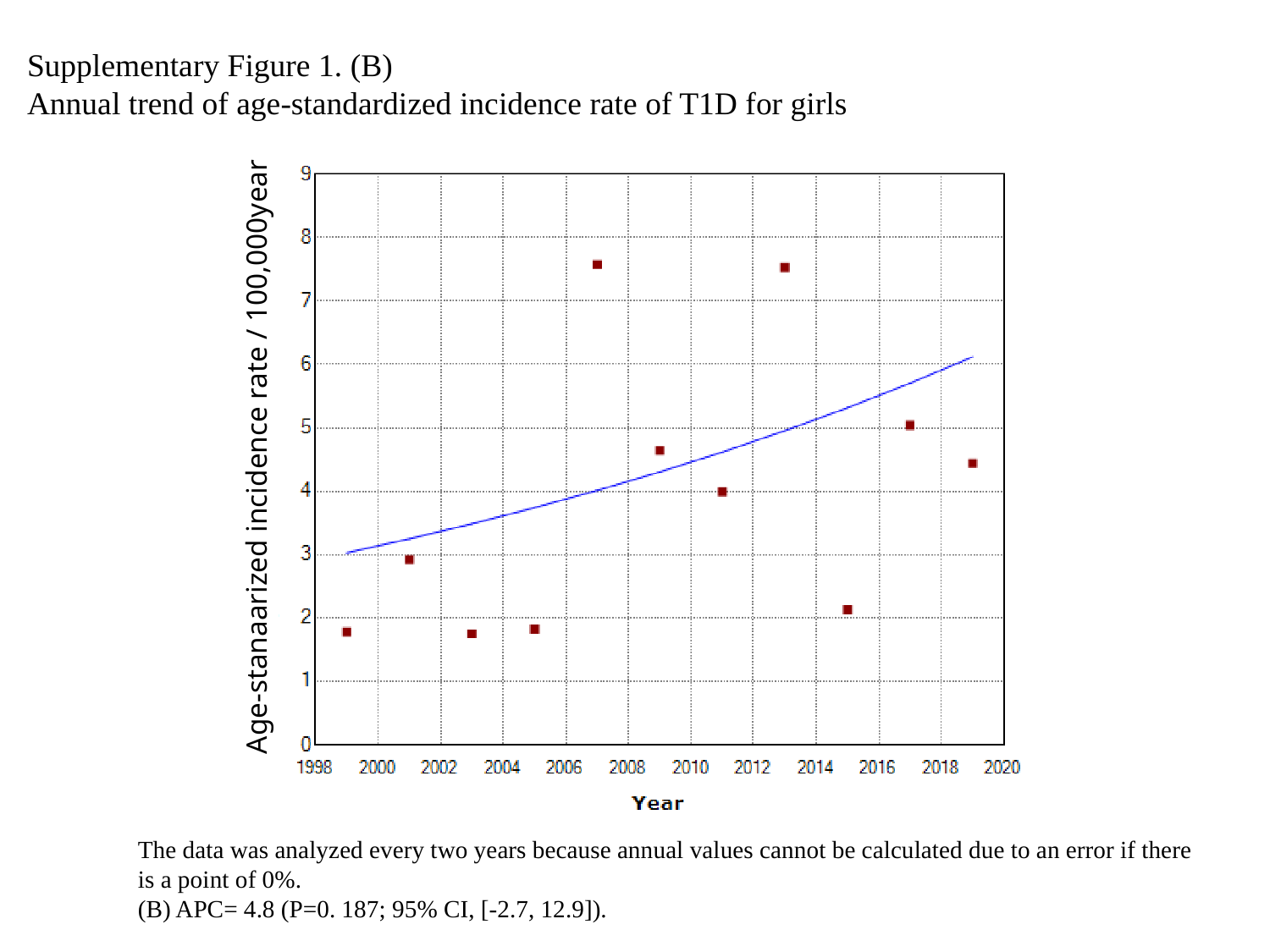

Supplementary Figure 1. (B)
Annual trend of age-standardized incidence rate of T1D for girls
Age-stanaarized incidence rate / 100,000year
The data was analyzed every two years because annual values cannot be calculated due to an error if there is a point of 0%.
(B) APC= 4.8 (P=0. 187; 95% CI, [-2.7, 12.9]).

## Slide 3
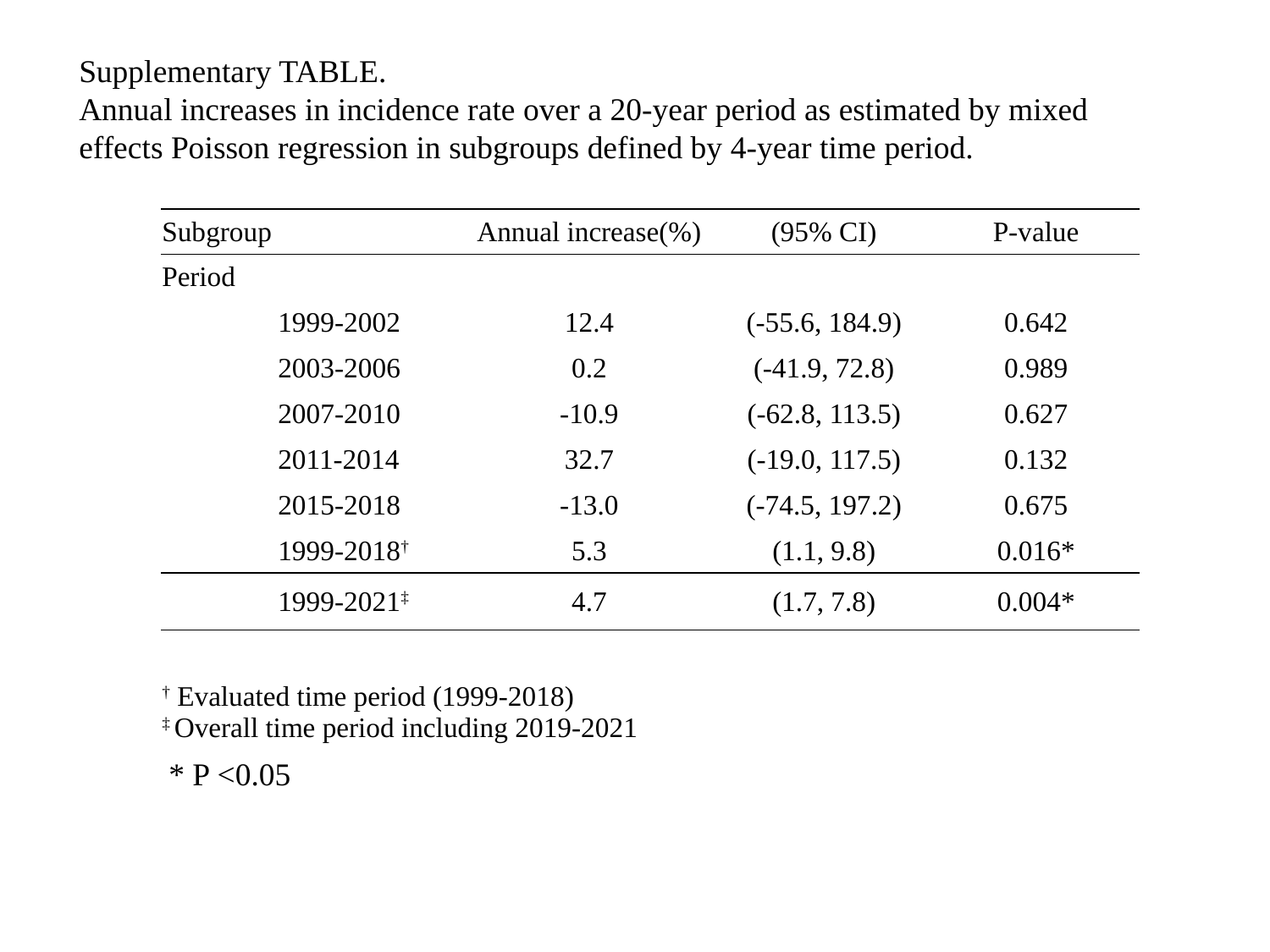

Supplementary TABLE.
Annual increases in incidence rate over a 20-year period as estimated by mixed effects Poisson regression in subgroups defined by 4-year time period.
| Subgroup | | Annual increase(%) | (95% CI) | P-value |
| --- | --- | --- | --- | --- |
| Period | | | | |
| | 1999-2002 | 12.4 | (-55.6, 184.9) | 0.642 |
| | 2003-2006 | 0.2 | (-41.9, 72.8) | 0.989 |
| | 2007-2010 | -10.9 | (-62.8, 113.5) | 0.627 |
| | 2011-2014 | 32.7 | (-19.0, 117.5) | 0.132 |
| | 2015-2018 | -13.0 | (-74.5, 197.2) | 0.675 |
| | 1999-2018† | 5.3 | (1.1, 9.8) | 0.016\* |
| | 1999-2021‡ | 4.7 | (1.7, 7.8) | 0.004\* |
| † Evaluated time period (1999-2018) ‡ Overall time period including 2019-2021 | | | | |
* P <0.05
